# Plasma sample based analysis of gastric cancer progression using targeted metabolomics

**DOI:** 10.1038/s41598-017-17921-x

**Published:** 2017-12-19

**Authors:** Sergio Lario, Maria José Ramírez-Lázaro, Daniel Sanjuan-Herráez, Anna Brunet-Vega, Carles Pericay, Lourdes Gombau, Félix Junquera, Guillermo Quintás, Xavier Calvet

**Affiliations:** 1 Fundació Parc Taulí, Institut Universitari Parc Taulí-UAB, Parc Tauli 1, 08208 Sabadell, Spain; 2 Digestive Diseases Service, Hospital de Sabadell, Institut Universitari Parc Taulí-UAB, Parc Tauli 1, 08208 Sabadell, Spain; 3 Centro de Investigación Biomédica en Red de Enfermedades Hepáticas y Digestivas (CIBERehd), Instituto de Salud Carlos III, Montorte de Lemos 3-5, 28029 Madrid, Spain; 4 Health and biomedicine, Leitat Technological Center, Baldiri Reixac 15, 08028 Barcelona, Spain; 5 Oncology Service, Hospital de Sabadell, Institut Universitari Parc Taulí-UAB, Parc Tauli, 1, 08208 Sabadell, Spain; 6 Unidad Analítica, Instituto de investigación sanitaria Hospital universitario y politécnico La Fe, Avda. Fernando Abril Martorell 106, 46026 Valencia, Spain

## Abstract

Gastric carcinogenesis is a multifactorial process described as a stepwise progression from non-active gastritis (NAG), chronic active gastritis (CAG), precursor lesions of gastric cancer (PLGC) and gastric adenocarcinoma. Gastric cancer (GC) 5-year survival rate is highly dependent upon stage of disease at diagnosis, which is based on endoscopy, biopsy and pathological examinations. Non-invasive GC biomarkers would facilitate its diagnosis at early stages leading to improved GC prognosis. We analyzed plasma samples collected from 80 patients diagnosed with NAG without *H. pylori* infection (NAG−), CAG with *H. pylori* infection (CAG+), PLGC and GC. A panel of 208 metabolites including acylcarnitines, amino acids and biogenic amines, sphingolipids, glycerophospholipids, hexoses, and tryptophan and phenylalanine metabolites were quantified using two complementary quantitative approaches: Biocrates AbsoluteIDQ®p180 kit and a LC-MS method designed for the analysis of 29 tryptophan pathway and phenylalanine metabolites. Significantly altered metabolic profiles were found in GC patients that allowing discrimination from NAG−, CAG+ and PLGC patients. Pathway analysis showed significantly altered tryptophan and nitrogen metabolic pathways (FDR P < 0.01). Three metabolites (histidine, tryprophan and phenylacetylglutamine) discriminated between non-GC and GC groups. These metabolic signatures open new possibilities to improve surveillance of PLGC patients using a minimally invasive blood analysis.

## Introduction

Gastric cancer (GC) is the fifth most common cancer worldwide^1^. Gastric carcinogenesis is a multistep and multifactorial process beginning with active chronic gastritis induced by *Helicobacter pylori* (*H. pylori*) infection^2,3^. The progression is often described *via* a sequence of events known as Correa’s cascade^4,5^, a stepwise progression from non-active gastritis (NAG), chronic active gastritis (CAG), precursor lesions of gastric cancer (PLGC: atrophy, intestinal metaplasia, dysplasia) and gastric adenocarcinoma (GC). GC diagnosis is based on endoscopy, biopsy and pathological examinations and prognosis is related to the stage of disease at diagnosis^6^. When diagnosed and resected, the 5-year survival rate reaches over 90% if the resection was performed at a very early stage. However, early stages of GC are often asymptomatic and for patients diagnosed at advanced stages, the 5-year survival rate is reduced down to 20%^7^. Because of that, the development of non-invasive biomarkers for the detection of early stages might lead to improved GC prognosis.

The metabolome has been defined as the set of metabolites synthesized by an organism contributing to its metabolic reactions in a particular physiological or developmental stage^8,9^. Metabolites are downstream products of the genome and proteome and reflect their interactions and the interaction with the environment, providing a direct and meaningful read out of the biochemical status of a system. Reprograming of pathways of nutrient acquisition and metabolism to meet bioenergetic, biosynthetic and redox demands of tumor cells is recognized as a hallmark of cancer^10–13^. Cancer metabolism is a very active field of research that has lead to the development of positron emission tomography (PET) for tumor imaging^14^, the identification of metabolic enzymes as drug targets^15^, oncometabolites^16^ and disease biomarkers for precision medicine^17^. However, metabolomics has been scarcely used in GC research. Urinary metabolomes from GC patients and healthy individuals and 30 pairs of matched tumor and normal stomach tissues were analyzed using ^1^H nuclear magnetic resonance (NMR) and ^1^H high-resolution magic angle spinning spectroscopy, respectively showing amino acid and lipid alterations in urine from GC patients consistent with changes observed in GC tissue^18^. Gas chromatography hyphenated to mass spectrometry and ^1^H-NMR has been used to profile urinary metabolites on urine samples to discriminate between GC patients and healthy volunteers^19,20^. Gas chromatography – high resolution time-of-flight mass spectrometric analysis of plasma samples from chronic active gastritis and GC patients showed different metabolic profiles associated to oxidative stress, and perturbed metabolism of amino acids and fatty acids^21^. Results obtained from the analysis of plasma samples from GC patients showed significant alterations in the plasma free amino acids profiles^22^. Another study showed significant differences in a subset of 17 PFAAs between GC patients and age-matched healthy controls. Besides, levels of 4 PFAAs showed dynamic alterations during the perioperative period in GC patients^23^. We recently published a study reporting changes in the plasma metabolic profiles through disease progression within the Correa’s cascade^24^. Using liquid chromatography – high resolution mass spectrometry, the study involved the untargeted analysis of samples collected from patients with NAG and no *H. pylori* infection (NAG−), CAG and *H. pylori* infection (CAG+), PLGC with and without *H. pylori* infection and GC. Results obtained allowed the identification of tryptophan and one of its metabolites (kynurenine) as discriminant metabolites of GC that could be attributed to indoleamine-2,3-dioxygenase (IDO) up-regulation leading to tryptophan depletion and kynurenine metabolites generation. Furthermore, phenylacetylglutamine was also classified as a discriminant metabolite. Results also indicated that the observed metabolic changes could not be attributted to differences in the distribution of *H. pylori* infection across the groups of patients.

The objective of the current study was to continue this line of research through the identification of a plasma metabolic pattern characteristic of GC through disease progression within the Correa’s cascade using quantitative metabolomics. We present results obtained from the analysis of plasma samples collected from 80 patients diagnosed with NAG−, CAG+, PLGC and GC using a combination of mass spectrometry-based methods for the quantification of a total of 208 metabolites including acylcarnitines, amino acids and biogenic amines, sphingolipids, glycerophospholipids, hexoses, tryptophan and phenylalanine metabolites. Multivariate analysis showed significantly altered metabolic profiles in GC patients that allowed their discrimination from NAG−, CAG+ and PLGC patients and pathway analysis showed significantly altered tryptophan and nitrogen metabolic pathways. These metabolic signatures open new possibilities to improve surveillance of PLGC patients using a fast and minimally invasive blood analysis.

## Materials and Methods

### Study population and sample collection

Outpatients referred to the Endoscopy Unit for evaluation of dyspepsia and patients with GC undergoing preoperative endoscopic ultrasound between February 2009 and February 2015 were asked to participate. Dyspeptic patients were contacted three weeks prior to the endoscopy. Volunteers providing informed consent were instructed to avoid antisecretory drugs within two weeks before the test. Exclusion criteria were: patients unable to stop antisecretory drugs, those who had received antibiotics within 4 weeks before the endoscopy and those with previous *H. pylori* treatment^24^. Before the endoscopy, a^13^[C]-urea breath test (UBiTest 100 mg, Otsuka Pharmaceutical Europe Ltd, UK) was administered. During endoscopy, antral biopsies for histology and for rapid urease test (JATROX HP test CHR Heim Arzneimittel GmbH, Germany) were obtained. Histological examination was evaluated by a pathologist specialized in digestive diseases. Each specimen was studied for the presence of *H. pylori*, chronic active gastritis, atrophy, intestinal metaplasia and presence of lymphoid follicles. Patients with atrophy and/or intestinal metaplasia were classified as PLGC. Patients with concordance of rapid urease test, urea breath test and histopathology (Giemsa staining) were considered *H. pylori* positive. Patients with all tests negative were considered uninfected^25^. After surgical resection, the GC was staged according to the TNM staging system. Plasma samples from GC patients were collected before the endoscopic ultrasound procedure for preoperative staging. Blood samples were collected into Vacutainer EDTA-K3 tubes (BD Biosciences, Spain). Plasma was prepared within an hour by centrifugation at 2400 *×* *g* for 10 min at room temperature and stored at −80 °C until analysis. The set of plasma samples analysed in this study were collected from 80 patients (19 NAG−, 20 CAG+, 21 PLGC and 20 GC) (see Table 1). Informed consent to be included in the study, or the equivalent, was obtained from all patients. The study was approved by the Ethics Committee of the Corporació Sanitària Parc Taulí (Institut Universitari Parc Taulí, Sabadell, Spain) (approval number 2014544) and all methods were performed in accordance with the relevant guidelines and regulations.

**Table 1:** Dyspeptic (NAG−, CAG+, PLGC) and GC patient’s clinical and demographic data.

	N	Age (std)	Sex (F/M)	Hp ( + /−)	GC stage (I/II/III/IV)
NAG−	19	43 (11)	13/7	0/19	—
CAG+	20	48 (11)	13/7	20/0	—
PLGC	21	52 (14)	12/9	10/10	—
GC	20	68 (12)	8/12	0/20	8/2/3/7

### Chemicals and reagents

Acetonitrile (LC-MS grade) was obtained from Fisher Scientific (Madrid, Spain) and formic acid (analytical grade) was purchased from Sigma Aldrich Quimica SA (Madrid, Spain). Water was Milli-Q grade from a Millipore purification system. Standards of 3-indoleacetonitrile (Kioto Encyclopedia of Genes and Genomes (KEGG) number C02938), quinolinic acid (C03722), aminophenol (C01987), 3-hydroxykynurenine (C03227), p-tyrosine (C0082), m-tyrosine (HMDB59720), o-tyrosine (HMDB06050), serotonin (C00780), 5-hydroxytryptophan (C00643), L-kynurenine (C00328), phenylalanine (C00079), hydroxyanthranillic acid (C00632), tryptophan (C00078), xanthurenic acid (C02470), tryptamine (C00398), kynurenic acid (C01717), N-acetylserotonin (C00978), phenylacetylglutamine (PAGN) (C04148), indole-3-acetamide (C02693), anthranillic acid (C00108), melatonin (C01598), 3-indoleacetic acid (KEGG C00954), tryptophol (00955), indolelactic acid (C02043) were obtained from Sigma Aldrich Quimica. N-formylkynurenine (C02700), 6-hydroxymelatonin (C05643), 4-chloro-kynurenine, 5-methoxytryptamine (C05659) and N-Formyl-N-acetyl-5-methoxykynurenamine (C05642) were purchased from Toronto Research Chemicals (Toronto, Canada). Deuterated internal standards melatonin-D_4,_ 5-hydroxytryptophan-D_4,_ L-kynurenine-D_4,_ indole-D_5_-3-acetamide, 4-chloro-kynurenine-^13^C_2_,^15^N, 6-hydroxymelatonin-D_4_, kynurenic acid-D_5_, PAGN-D_5_, phenylalanine-D_5_, serotonin-D_4_, tryptamine-D_4_, tryptophan-D_5_, xanthurenic acid-D_4_, were obtained from Toronto Research Chemicals. Phenylalanine-D_5_ was purchased from Cambridge Isotope Laboratories (Andover, USA).

### Metabolomic analysis

EDTA-plasma samples stored at −80 °C were thawed on ice. Sample analysis was carried out using two quantitative approaches: i) the Biocrates’s AbsoluteIDQ® p180 kit (Biocrates Life Sciences AG, Innsbruck, Austria) and ii) a novel method for the quantification of 29 analytes including tryptophan and phenylalanine metabolites. Sample analysis using the Biocrates’s AbsoluteIDQ® p180 kit was carried out according to the manufacturer’s guidelines on an Acquity-Xevo TQS (Waters, Milford, USA) system equipped with an electrospray ionization source. The kit is designed for the measurement of a total of 40 acylcarnitines, 42 amino acids and biogenic amines, 15 sphingolipids, 90 glycerophospholipids and 1 group of metabolites (sum of hexoses). For the quantification of 29 tryptophan metabolites, phenylalanine, p-tyrosine, m-tyrosine, o-tyrosine and phenylacetylglutamine using the second method, aliquots of 50 µL of plasma samples were placed into 1.5 mL eppendorf tubes and 150 µL of cold CH_3_CN were added for protein precipitation. Then, samples were vortexed for 15 s and subsequently centrifuged at 15000 × *g* for 10 min at 4 °C. The supernatants were transferred to clean tubes and evaporated in a Thermo SPD121P SpeedVac concentrator (Waltham, MA USA). The residues were reconstituted in 50 µL of internal standards solution of hydroxytryptophan-D_4,_ L-kynurenine-D_4,_ indole-D_5_-3-acetamide, 4-chloro-kynurenine-^13^C_2_,^15^N, 6-hydroxymelatonin-D_4_, kynurenic acid-D_5_, PAGN-D_5_, phenylalanine-D_5_, serotonin-D_4_, tryptamine-D_4_, tryptophan-D_5_, xanthurenic acid-D_4_ and phenylalanine-D_5_ (900 nM each). Afterwards, samples were centrifuged at 15000 × *g* for 5 min at 4 °C. Finally, the supernatants were transferred to a 96-well plate for analysis. A diluted sample (dilution factor: 20) was prepared to ensure that metabolites typically present at higher concentrations in samples (e.g. tryptophan) fall within the linear range. UPLC-MS/MS analysis was carried out on an Acquity-Xevo TQS system.

### Analysis of tryptophan and phenylalanine metabolites in plasma samples

**Figure 1:**
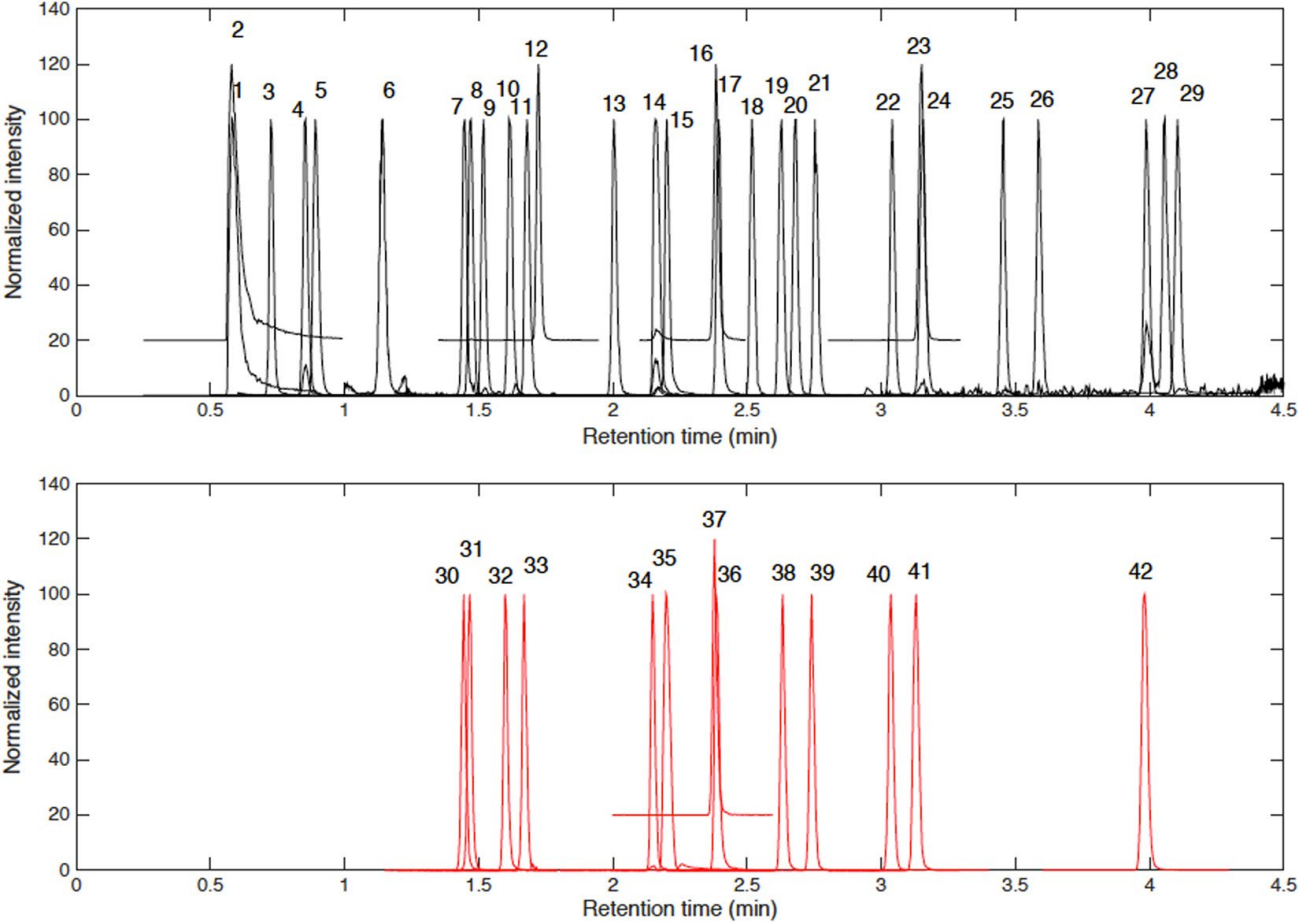
Typical chromatograms of the Trp and Phe metabolites extracted from the analysis of spiked plasma sample. 1: 3-indoleacetonitrile; 2: quinolinic acid; 3: aminophenol; 4: 3-hydroxykynurenine; 5: p-tyrosine; 6: m-tyrosine; 7: serotonin; 8: 5-hydroxytryptophan; 9: o-tyrosine; 10: kynurenine; 11: phenylalanine; 12: N-formylkynurenine; 13: hydroxyanthranillic acid; 14: tryptophan; 15: xanthurenic acid; 16: tryptamine; 17: kynurenic acid; 18: 5-methoxytryptamine; 19: 4-chlorokynurenine; 20: N-acetylserotonin; 21: phenylacetylglutamine; 22: 6-hydroxymelatonin; 23: indole-3-acetamide; 24: anthranillic acid; 25: formyl-acetylmethoxykynurenamine; 26: indolelactic acid; 27: melatonin; 28: 3-indoleacetic acid; 29: tryptophol; 30: serotonin-D_4_; 31: 5-hydroxytryptophan-D_4_; 32: kynurenine-D_4_; 33: phenylalanine-D_5_; 34: tryptophan-D_5_; 35: xanthurenic acid-D_4_; 36: kynurenic acid-D_5_; 37: tryptamine-D_4_; 38: 4-chloro-kynurenine-^13^C_2_,^15^N; 39: phenylacetylglutamine-D_5_; 40: 6-hydroxymelatonin-D_4_; 41: indole-3-acetamide-D_5_; 42: melatonin-D_4_.

Samples were analysed using an Acquity HSS T3 C_18_ (100 × 2.1 mm, 1.8 µm) column. Mobile phases were H_2_O (0.1% v/v HCOOH) (A) and (0.1% v/v HCOOH) CH_3_CN (B). The gradient elution was as follows: phase B was held 2% from 0 to 0.5 min, then increased linearly to 45% over the following 5 min. Then phase B was increased to 90% in 0.2 min followed by a fast return to initial conditions between 5.7 and 6 min, which were held for 1.5 min for column re-equilibration. Injection volume, flow rate and column temperature were set at 3 µL, 550 µL/min and 55 °C, respectively. Autosampler temperature was set at 6 °C during sample analysis. Electrospray ionization was carried out using the following conditions: capillary 2.9 kV, cone 25 V, source temperature 120 °C, desolvation temperature 395 °C, N_2_ cone and desolvation gas flow rates were 150 and 800 L/h, respectively. In spite of the high structural similarity among metabolites, their chromatographic resolution (see Fig. 1) and the main figures of merit of the UPLC-MS/MS method summarized in Table S1 (i.e. retention time, limit of detection, linear range, repeatability and accuracy as mean recovery in spiked samples) provided satisfactory results with limits of detection in the nM range and recoveries in spiked samples in the 70–130% range.

### Statistical analysis

Statistical analysis included an initial data clean up step to increase the reliability of the results. Accordingly, metabolites analyzed using the Biocrates AbsoluteIDQ® p180 kit were excluded from further analysis if either (i) the relative standard deviation of three replicates of a quality control (QC) sample was higher than 25%, or (ii) the median concentration of the metabolite over all samples included in each group of samples (i.e. NAG, CAG+, PLGC or GC) was lower than the correspoding lower limit of quantification calculated according to the Biocrate’s guidelines. Metabolites analysed by the UPLC-MS/MS method for the analysis of tryptophan pathway and phenylalanine metabolites were excluded from further analysis if the number of missing values was higher than 20% in each group of samples (i.e. NAG−, CAG+, PLGC or GC). Trytophan, phenylalanine, p-tyrosine, kynurenine and serotonin concentrations provided by both methods were, as expected, highly correlated and only those obtained using the Biocrates approach were retained for further analysis. The final data set comprised concentrations of 83 metabolites.

Univariate two-sides *t*-test were carried out to evaluate the null hypothesis that the concentrations of the metabolites in two groups come from independent random samples from normal distributions with equal means, without assuming that the populations also have equal variances. Metabolites with FDR^26^ adjusted p-values < 5% were selected as discriminants. Principal component analysis (PCA) and partial least squares – discriminant analysis (PLS-DA) were carried out using autoscaled data to adjust for the differences in fold differences between the metabolites^27^. The selection of the optimal number of latent variables of PLS-DA models and the estimation of their classification accuracy was carried out by *k*-fold cross validation (*k*-fold CV). In this work a *k* = 5 was selected as a compromise between the amount of available training data for model fitting (higher *k* values may lead to overly optimistic estimates) and the higher variance of the CV-estimates provided by lower *k* values. Besides, to reduce the impact of the random split of CV-subsets, the mean values of the estimates obtained after 20 random 5-fold CV were used. The classification accuracy, the area under the receiver operating curve (AUROC) as well as the sensitivity, selectivity and negative and positive predictive values (NPV and PPV, respectively) were employed as figures of merit. The statistical significance of the CV figures of merit was assessed by permutation testing, where null distributions were estimated by 1000 random permutations of the class labels and then, *p*-values were computed as the fraction of permuted statistics that are at least as extreme as the test statistic obtained using the original class labels^28^.

### Software

Data acquisition was carried out using MassLynx (Waters) software. Metabolite concentrations were calculated using MassLynx and MetIQ (Biocrates) software. PCA and PLS-DA were carried out using PLS Toolbox 8.0 (Eigenvector Research Inc., Wenatchee, USA) and in-house written MATLAB (Mathworks Inc., Natick, MA, USA) scripts. Pathway analysis was carried out with MetaboAnalyst 3.0 (McGill University, Canada)^29^. The datasets generated during and/or analysed during the current study are available from the corresponding author on reasonable request.

## Results

### Association among metabolic profiles and GC progression group

**Figure 2:**
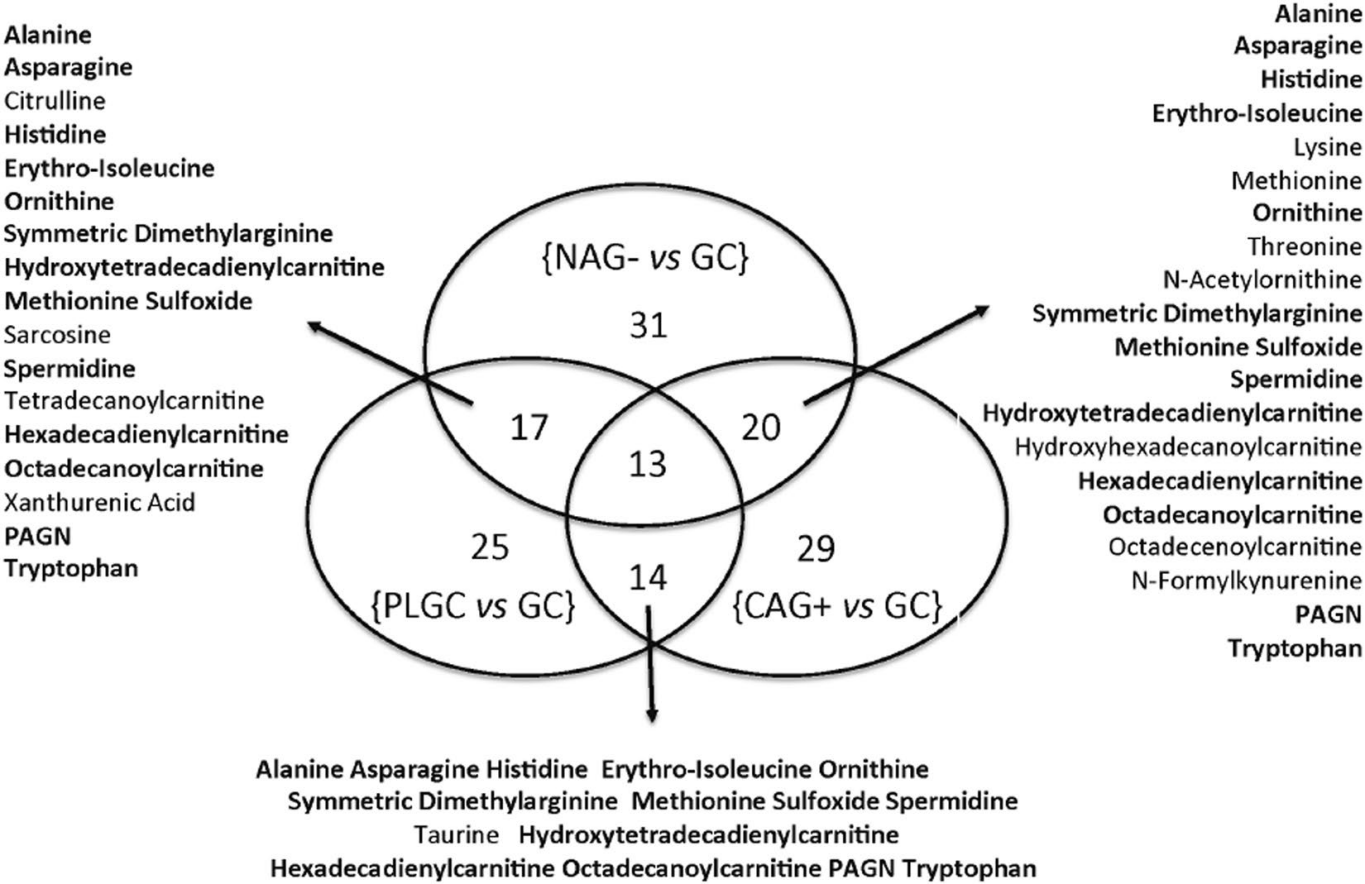
Discriminant metabolites. Venn diagram showing the metabolites selected as highly discriminant (VIP > 1) in the NAG− *vs* GC, CAG + *vs* GC and PLGC *vs* GC models. Metabolites commonly selected in the three models are highlighted in bold.

**Figure 3:**
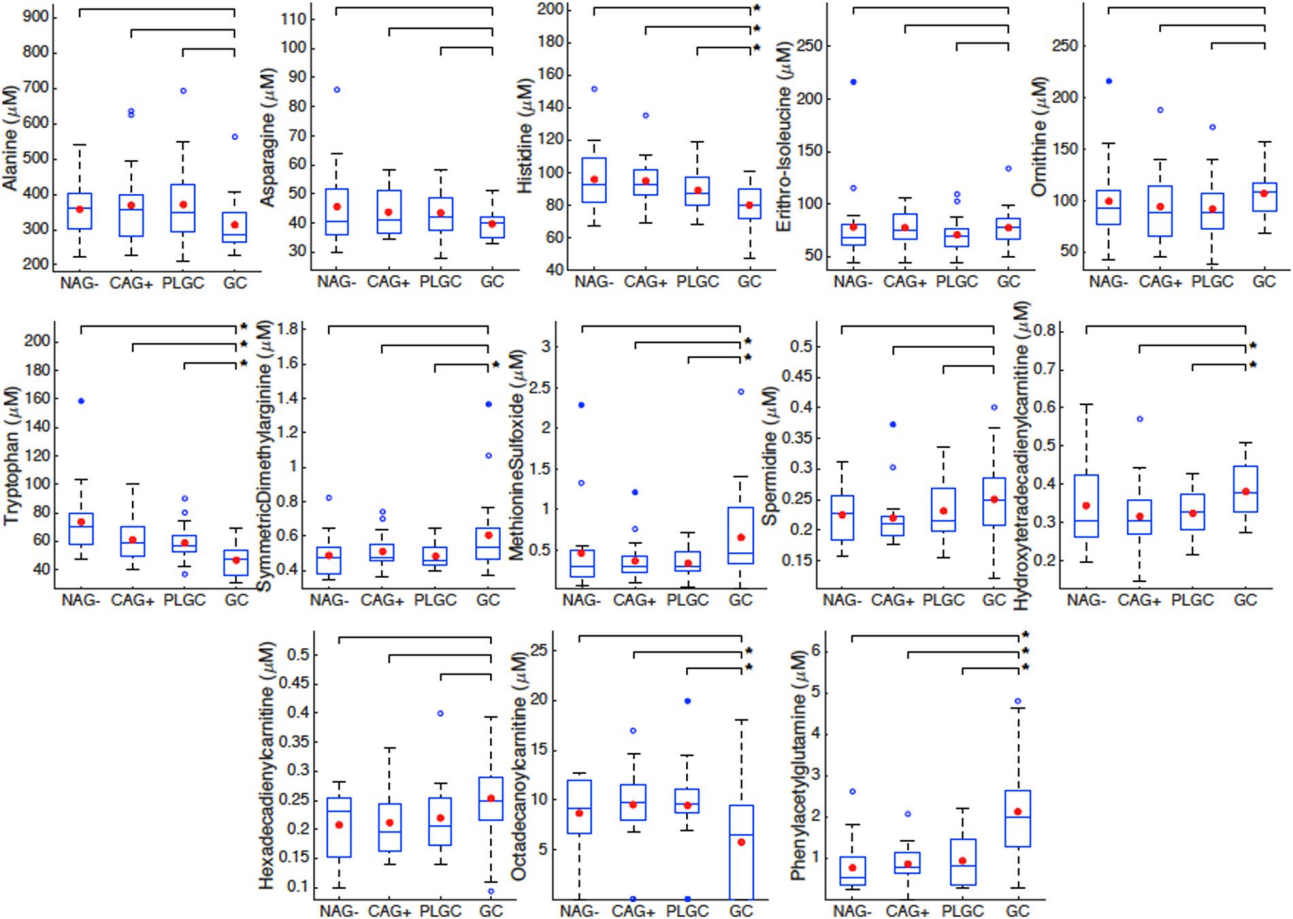
Metabolite concentrations. Boxplots of the metabolites commonly selected as highly discriminant (VIP > 1) in the three PLS-DA models between GC and NAG, CAG+ and PLGC groups. Note: *indicates metabolite with FDR adjusted t-test *p*-value < 5% between GC and non-GC groups.

A PCA model was calculated for the identification of outliers and to obtain an initial overview of the data. The PC1 *vs* PC2 scores plot obtained from the PCA model of the data set explaining 22% of the total variance showed a high overlap of NAG−, CAG+, PLGC or GC samples (see Figure S1). No clustering among the four groups was observed using higher PCs (data not shown). Then, a supervised multi-class PLS-DA model was build to discriminate among the four groups of patients. PLS-DA scores plot obtained is depicted in Figure S1 indicated a clustering of GC samples and a high overlap of NAG−, CAG+ and PLGC groups. Cross validation of a PLS-DA build for the discrimination of the four groups of samples showed a statistically significant discrimination between GC and NAG−, CAG+ and PLGC samples (AUROC = 0.86, *p*-value < 0.05), in agreement with the PLS-DA scores plot depicted in Figure S1. Besides, the previously observed overlapping of NAG−, CAG+ and PLGC metabolic profiles lead to a non-significant discrimination among these groups (AUROC *p*-values ⨠ 0.05). Univariate t-test did not identify metabolites potentially discriminant between NAG− and CAG+ or between CAG+ and PLGC. The comparison of the levels of GC and the set of NAG−, CAG+ and PLGC samples identified tryptophan, phenylacetylglutamine and histidine as a potentially discriminant metabolites. Besides, phenylacetylglutamine and formylkynurenine discriminated between PLGC and GC and NAG− groups, respectively. However, FDR correction is conservative and increases the risk of introducing false negatives (i.e. type II error)^30^. To facilitate the interpretation of the statistical significance of the differences among groups observed by PCA and the selection of the most discriminant metabolites of GC, six independent binary PLS-DA models were calculated in which the four classes were compared pairwise. Results from the evaluation of the classification performance of the six models by cross validation are summarized in Table 2. The identification of the most relevant metabolites responsible for the observed discrimination of GC samples was carried out using the variable influence in the projection (VIP) scores from the previously developed binary PLS-DA models^31^. Figure 2 highlights the metabolites selected as highly discriminant (VIP > 1) in the NAG− *vs* GC, CAG+ *vs* GC and PLGC *vs* GC models. The set of 47 metabolites with VIP > 1 included 16 amino acids, 10 biogenic amines and modified amino acids, 13 acylcarnitines, 7 tryptophan metabolites and a phenylalanine metabolite (phenylacetylglutamine). Among them, a total of 13 metabolites were commonly selected in the three models including acylcarnitines (hydroxytetradecadienylcarnitine and octadecanoylcarnitine), amino acids (alanine, asparagine, histidine, erithro-isoleucine and tryptophan), biogenic amines (symmetric dimethylarginine, methionine sulfoxide, ornithine and spermidine) and phenylacetylglutamine. Figure 3 shows boxplots of their concentrations in the four groups of patients.

**Table 2:** Evaluation of the discrimination among NAG−, CAG+, PLGC and GC metabolic profiles by PLS-DA using cross validated accuracy (i.e. % correctly classified samples), AUROC, sensitivity, specificity, PPV and NPV estimates.

Model	Latent variables	Accuracy (*p-*value)^1^	AUROC (*p-*value)	Sensitivity (*p-*value)	Specificity (*p-*value)	PPV (*p-*value)	NPV (*p-*value)
NAG- *vs* CAG+	2	0.41 (>0.05)	0.43 (>0.05)	0.40 (>0.05)	0.42 (>0.05)	0.42 (>0.05)	0.40 (>0.05)
NAG- *vs* PLGC	1	0.56 (>0.05)	0.82 (>0.05)	0.38 (>0.05)	0.56 (>0.05)	0.60 (>0.05)	0.60 (>0.05)
CAG + *vs* PLGC	1	0.53 (>0.05)	0.49 (>0.05)	0.57 (>0.05)	0.50 (>0.05)	0.54 (>0.05)	0.53 (>0.05)
NAG− *vs* GC	2	0.77 (0.002)	0.83 (0.002)	0.80 (0.002)	0.74 (0.004)	0.76 (0.004)	0.78 (0.004)
CAG + *vs* GC	2	0.80 (0.002)	0.81 (0.002)	0.75 (0.008)	0.85 (0.002)	0.83 (0.002)	0.77 (0.006)
PLGC *vs* GC	2	0.68 (0.016)	0.74 (0.006)	0.70 (0.01)	0.67 (0.04)	0.70 (0.03)	0.70 (0.01)

^1^ *p*-values were computed by permutation testing as the fraction of permuted statistics that are at least as extreme as the test statistic obtained using the original class labels.

Within-class differences in the metabolic profiles according to the cancer staging (TNM I + II *vs* TNM III + IV) were also evaluated using PLS-DA. Due to the limited number of samples, leave-one out-CV was used in this case for model optimizaton and for the evaluation of the significance of the discrimination according to the staging. Results obtained using 2 latent variables showed no statistically significant difference according to the cancer stage (permutation test *p*-values: classification accuracy = 0.45, *p*-value > 0.05; AUROC = 0.31, *p*-value > 0.05; sensitivity = 0.40, *p*-value>0.05; selectivity = 0.50, *p*-value > 0.05).

### Pathway analysis

**Figure 4:**
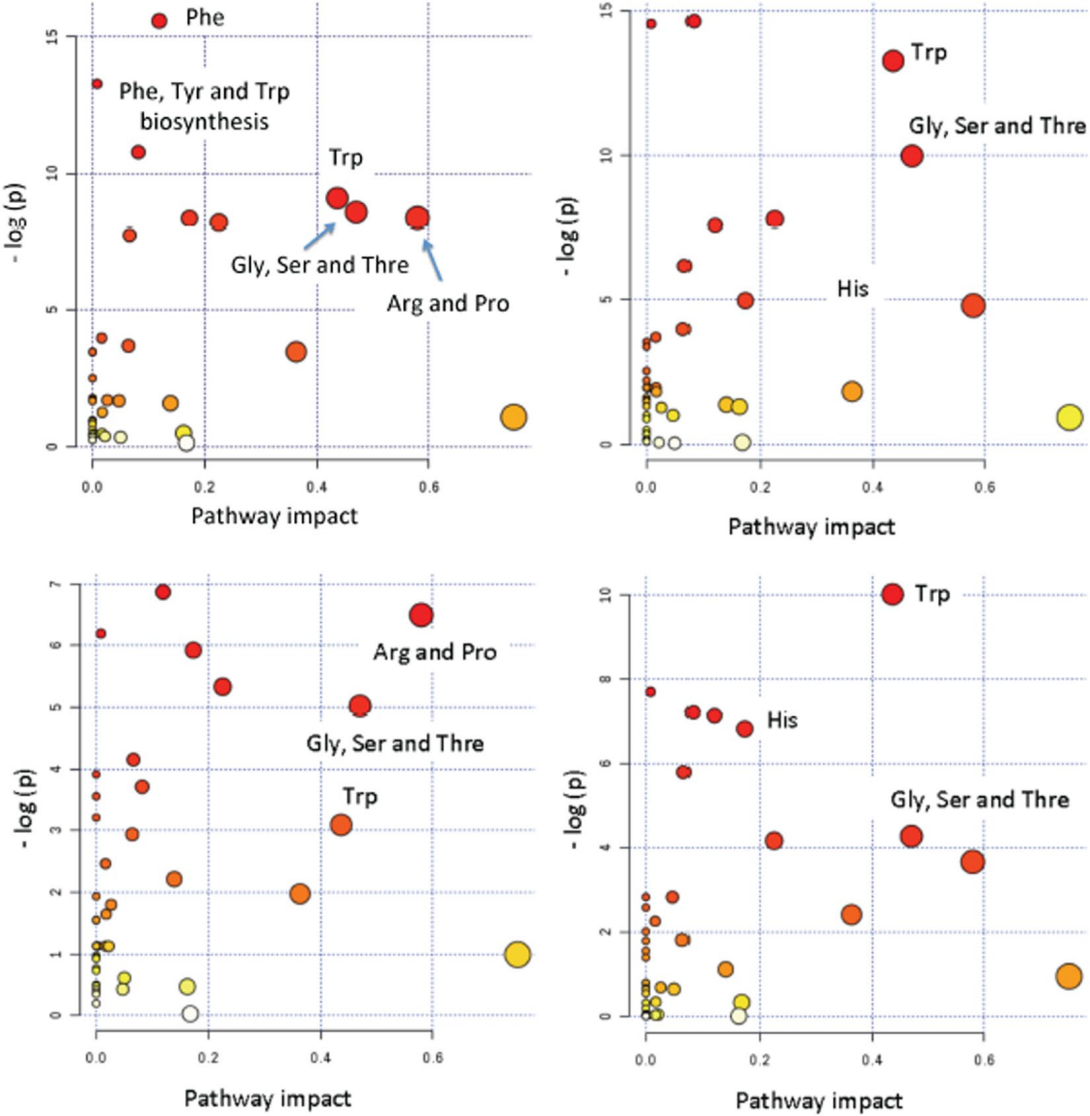
Pathway analysis. Results from pathway analysis using autoscaled data after quantile normalization, a global test for enrichment analysis and a relative-betweeness centrality topology analysis to measure the relative importance of each metabolite in a given pathway (clockwise from top left) GC *vs* non-GC; GC *vs* NAG−; GC *vs* CAG + ; GC *vs* PLGC.

**Figure 5:**
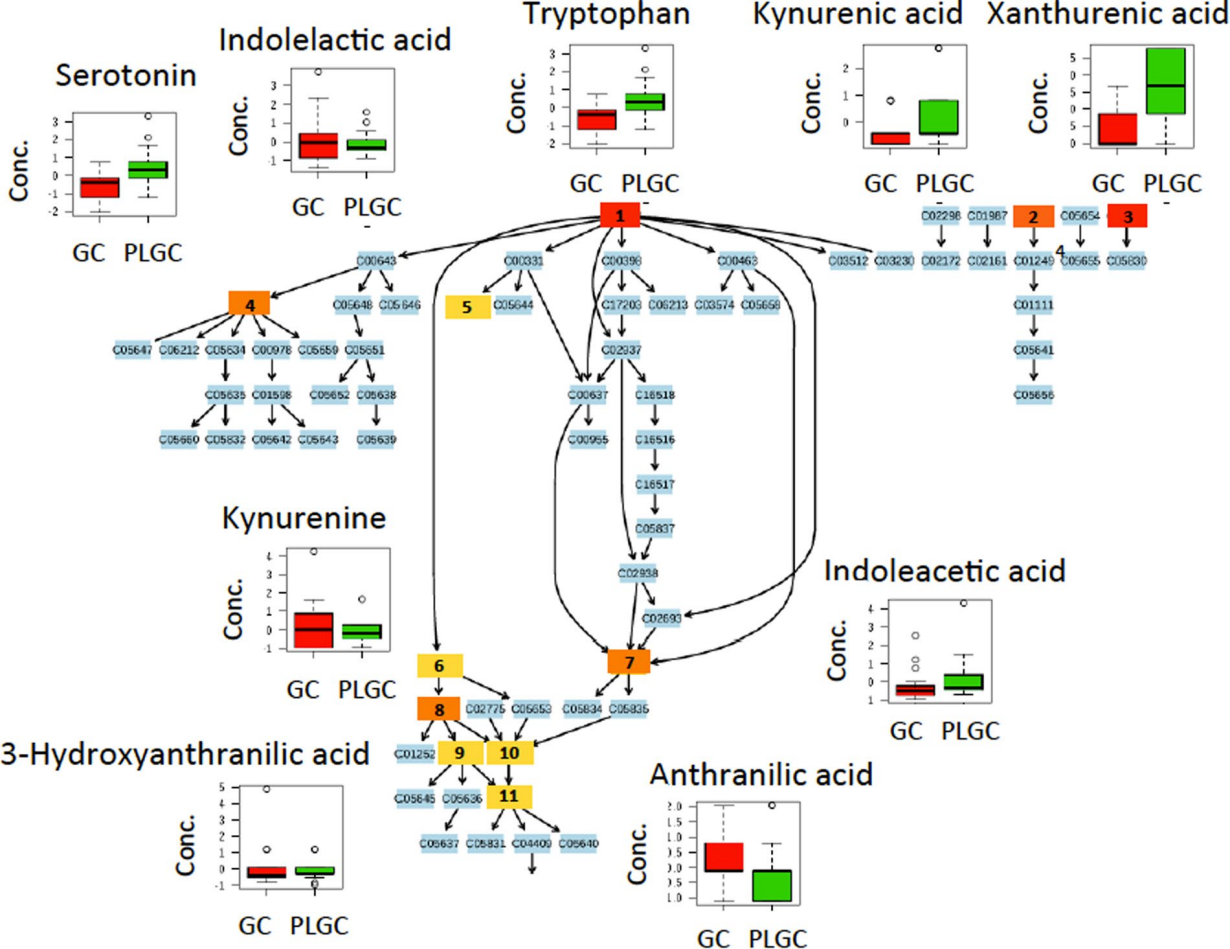
Overview of differences in the relative concentrations of detected metabolites in GC and PLGC groups in a subset of the trypophan phatway. Note: 1) tryptophan; 2) kynurenic acid; 3) xanthurenic acid; 4) serotonin; 5) indolelactic acid; 6) formylkynurenine; 7) indoleacetic acid; 8) kynurenine; 9) 3-hydroxykynurenine; 10) anthranilic acid; 11) 3-hydroxyanthranilic acid.

Pathway analysis was use to extract biological information within relevant networks of metabolic pathways. Pathway analysis integrates metabolite set enrichment analysis and pathway topology analysis^11^. Using quantile normalization and autoscaling as data pretreatment, the pathway enrichment and topology analysis were carried out using a global test and a relative betweenness centrality measure, respectively. Pathways analysis of the differences of GC and NAG−, CAG+ and PLGC groups independently and between GC and the set of ‘non-GC’ groups (i.e. NAG−, CAG+ and PLGC) was performed using MetaboAnalyst^11^. Five acylcarnitines without matching KEGG ID were excluded from the analysis. Results obtained are depicted in Fig. 4, where the color and the size of each circle indicates its *p*-value and pathway impact value, respectively. Tryptophan metabolism, as well as phenylalanine, nitrogen, arginine, proline, alanine and histidine metabolisms and phenylalanine, tyrosine and tryptophan biosynthesis pathways were found significantly altered (false discovery rate (FDR) adjusted *p*-value < 0.05) (see Table 3). As a relevant example, Fig. 5 depicts relative concentrations of nine metabolites in the tryptophan pathway quantified in plasma samples from GC and PLGC patients.

**Table 3:** List of significantly altered pathways in GC *vs* non-GC, NAG−, CAG+ and PLGC samples from pathway analysis.

Pathway name	Match status	FDR GC *vs* non GC	FDR GC *vs* NAG	FDR GC *vs* CAG+	FDR GC *vs* PLGC
**Trp metabolism**	11/79	2 10^−4^	2 10^−5^	>0.05	2 10^−3^
Phe metabolism	3/45	7 10^−6^	1 10^−5^	0.03	8 10^−3^
Arg and Pro metabolism	13/77	1 10^−3^	0.04	0.03	>0.05
Nitrogen metabolism	11/39	7 10^−5^	2 10^−5^	0.03	8 10^−3^
Phe, Tyr and Trp biosynthesis	4/27	8 10^−4^	1 10^−5^	>0.05	8 10^−3^
Gly, Ser and Thr metabolism	6/48	1 10^−3^	4 10^−4^	0.05	>0.05
Aminoacyl-tRNA biosynthesis	18/75	1 10^−3^	3 10^−3^	0.04	>0.05
Beta-Alanine metabolism	3/28	2 10^−3^	0.01	>0.05	0.02
Histidine metabolism	4/44	1 10^−3^	0.04	0.03	9 10^−3^

Note: data was autoscaled after quantile normalization. A global test for enrichment analysis and a relative-betweeness centrality topology analysis was employed to measure the relative importance of each metabolite in a given pathway.

## Discussion

Metabolic phenotypes are the result of a combination of genomic, transcriptomic and proteomic conditions and their interaction with the environment. Besides, metabolites are produced from other metabolites leading to a high level of interdepence characteristic of metabolomic data^32^. As a result of this complexity, multivariate models are typically needed to summarize the information contained and reveal underlying trends in the data. PCA is possibly the most widely used algorithm for unsupervised pattern recognition in metabolomics. PCA provides an unbiased dimensionality reduction that provides a visual representation of the data structures as the distances among observations in the score space can be related to the pattern summarized by the model^33^. PCA scores plot depicted in Figure S1 did not reveal a specific structure related to GC progression because the within-group variation was not sufficiently lower than between-group variation^34^. Nonetheless, the PCA model was used to assess the absence of outlying samples based on their relative position to the 95% confidence limit.

We expect relationships among metabolic variables associated to the phenotype and so, besides assessing the significance of each metabolite independently, multivariate discriminant models were calculated. Figure S1 shows the scores plot obtained from the PLS-DA analysis of the plasma samples. PLS-DA scores plots should not be used for the evaluation of the between-class differences because they provide overly optimistic results and may falsely suggest excellent separation among groups^9,35^. However, it provided valuable information on the presence of within-class clusters^15^. Results depicted in Figure S1 showed that NAG−, CAG+ and PLGC samples together in the PLS-DA scores space and GC samples clustered separately. Besides, no sub-clusters were initially identified among NAG−, CAG+, PLGC groups or within GC samples according to TNM stage. This initial observation was in agreement with results from the evaluation of the discriminant performance of PLS-DA models showing a statistically significant discrimination between GC and NAG−, CAG+ and PLGC samples (AUROC = 0.86, p-value < 0.05) and a non statistically significant discrimination among NAG−, CAG+ and PLGC metabolic profiles (AUROC p-values ⨠ 0.05). Likewise, results summarized in Table 2 from six independent binary PLS-DA models where the four classes were compared pairwise (i.e. NAG− *vs* CAG+, NAG− *vs* PLGC, NAG− *vs* GC, CAG+ *vs* PLGC, CAG+ *vs* GC and PLGC *vs* GC) only indicated a statistically significant (*p*-values < 0.05) discrimination between GC and NAG−, CAG+ and PLGC groups.

Results from pathway analysis of the four considered models depicted in Fig. 4 revealed a number of significantly altered pathways. Comparing GC *vs* non-GC groups, the pathways of tryptophan metabolism, as well as phenylalanine, nitrogen, arginine, proline, alanine and histidine metabolisms and phenylalanine, tyrosine and tryptophan biosynthesis pathways were significantly altered after multiple testing corrections (FDR *p*-value < 0.05) (see Table 3). Among them, tryptophan metabolism, as well as phenylalanine, nitrogen, alanine and histidine metabolisms and phenylalanine, tyrosine and tryptophan biosynthesis pathways were also significantly altered after multiple testing corrections (FDR *p*-value < 0.05) when GC and PLGC metabolic profiles were compared.

The relative decrease of tryptophan and increase of phenylacetylglutamine in GC patients (see Figs 3 and 5) were in agreement with results found in a previous untargeted metabolomic study^24^ and with the abovementioned alteration of the tryptophan pathway, supporting the robustness of the analysis. Besides, statistically significant (t-test *p*-value < 0.05) lower levels of kynurenic and xanthurenic acids and higher levels of serotonin and anthranillic acid were also observed in GC compared to PLGC patients (see Fig. 5). Immune dysregulation is a key event for tumor evasion of the host immune system. Lower tryptophan concentrations could be due to upregulated expression of the Trp-metabolizing enzymes IDO and IDO2 and the liver enzyme tryptophan dioxygenase (TDO)^18,19,36^. IDO and IDO2 control the Trp catabolism signaling pathway generating kynurenine and other downstream catabolites that can modulate T-cell immunity. Tryptophan depletion by TDO in IDO-negative tumors also induces signaling events in T cells, leading to anergy and apoptosis^37^. Enhanced IDO expression in *H. pylori* infected human gastric mucosa also modulates Th1/Th2 and Th17 pathways^38^. Higher concentrations of phenylacetylglutamine in GC patients could indicate a deregulation of the phenylalanine or glutamine metabolism. Phenylacetylglutamine is also a known microbial metabolite^20^ and so, observed changes of its plasmatic levels could either be attributed to the microbial or host metabolism or their interaction. The relative decrease in alanine, asparagine and histidine levels in GC patients shown in Fig. 3 was in agreement with previous studies in which urine^21^ and plasma^22^ levels from healthy volunteers and GC patients were compared and it might be due to more active nucleic acid metabolism in tumor cells. Histidine concentrations are also regulated by the enzime histidine decarboxylase (HDC) that converts L-histidine to histamine. Histamine was excluded during the initial data clean up and its variation as a function of disease progression could not be assessed. Concentrations of symmetric dimethylarginine and citrulline were increased in GC (see Fig. 3). Citrulline increased concentrations observed in GC compared to non-GC patients disagree with previously reported lower concentrations in GC patients compared to a control population^22^. GC patients showed lower concentrations (t-test, *p*-value < 0.05) of arginine than NAG and CAG+ groups, but GC and PLGC showed comparable levels. Previous results have shown low plasma arginine concentrations in cancer patients^39^. Symmetric dimethylarginine is a structural isomer of symmetric dimethylarginine produced as protein turnover that may have an indirect effect on endothelial nitric oxide synthase activity by interfering celullar L-arginine uptake. Higher concentrations of methionine sulfoxide were also observed in the GC group (see Fig. 3). Unbalanced production of free radicals (e.g. reactive oxigen species, ROS) leads to the oxidation of amino acids and free amino acids protein residues. Methionine is a target of ROS leading to the production of methionine sulfoxide^40^. However, oxidation of methionine residues as a consequence of increased oxidative stress in cancer cells can be enzymatically reversed to restore protein function by methionine sulfoxide reductase.

GC is a highly heterogenous disease. Further research focusing on the analysis of longitudinal trajectories of metabolic biomarkers will be critical to assess sources of short term variability and inter-individual variability, specially in PLGC patients progressing to GC to improve the robustness of the biomarker and its translation into clinical practice.

## Conclusion

The use of targeted metabolomics for the identification of GC biomarkers seems to be promising and support results obtained in a previous untargeted study. Results obtained showed significantly altered metabolomic profiles in GC patients that allowed their discrimination from NAG−, CAG+ and PLGC patients. Pathway analysis showed nitrogen and tryptophan metabolism significantly altered. Further studies are needed to fully map the relationship between changes in the phenylalanine and tryptophan metabolisms and oxidative stress with GC development. Besides, extending the study to a healthy group could lead to potential biomarker candidates that would need further validation and consolidation studies. Nonetheless, results obtained open new possibilities to improve surveillance of PLGC patients using a minimally invasive blood analysis that would facilitate GC diagnosis at early stages leading to improved prognosis.

## Electronic supplementary material

Supplementary material
