# Supplementary material for: Plasma sample based analysis of gastric cancer progression using targeted metabolomics
Source: Sci Rep. 2017 Dec 19;7:17774. doi: 10.1038/s41598-017-17921-x (PMC5736578; doi:10.1038/s41598-017-17921-x)
Supplement: Supplementary file 1 — Supplementary material [file 41598_2017_17921_MOESM1_ESM.pdf]

Plasma sample based analysis of gastric cancer progression using targeted metabolomics

Sergio Lario[a,b,c], Maria José Ramírez-Lázaro[b,c*], Daniel Sanjuan-Herráez[d], Anna Brunet-Vega[a,e], Carles Pericay[e], Lourdes Gombau[d], Félix Junquera[b,c], Guillermo Quintás[d,f**], Xavier Calvet[b,c]*

[a]Fundació Parc Taulí, Institut Universitari Parc Taulí-UAB, Parc Tauli 1, 08208 Sabadell, Spain

[b]Digestive Diseases Service, Hospital de Sabadell, Institut Universitari Parc Taulí-UAB, Parc Tauli 1, 08208 Sabadell, Spain

[c]Centro de Investigación Biomédica en Red de Enfermedades Hepáticas y Digestivas (CIBERehd), Instituto de Salud Carlos III, Montorte de Lemos 3-5, 28029 Madrid, Spain

[d]Health and biomedicine, Leitat Technological Center, Baldiri Reixac 15, 08028 Barcelona, Spain

[e]Oncology Service, Hospital de Sabadell, Institut Universitari Parc Taulí-UAB, Parc Tauli, 1, 08208 Sabadell, Spain

[f]Unidad Analítica, Instituto de investigación sanitaria Hospital universitario y politécnico La Fe, Avda. Fernando Abril Martorell 106, 46026 Valencia, Spain

* These authors contributed equally to this work.

***** corresponding author: gquintas@leitat.org Phone: +34 664819758

Table S1. Main UPLC-MS/MS acquisition parameters and analytical figures of merit for the quantificacion of 29 metabolites and 13 internal standards.

Metabolite	RT (min)	ESI	MRM Quant.	Cone (V)	CE (eV)	MRM Qual.	Cone (V)	CE (eV)	LOD (nM)	LR (nM)	RSD (%)	Recovery (%)	IS
3-Indoleacetonitrile	0.58	+	157.1>79	10	5	157.1>64	10	25	20	40-5000	4	84	Serotonin-D_4
Quinolinic acid	0.60	+	168.0>129	20	10	168.0>90	20	15	20	40-5000	4	77	Serotonin-D_4
Aminophenol	0.73	+	110.1>92.0	35	15	110.1>93.0	35	10	2.5	10-5000	6.6	128	5OHTrp-D_4
3-hydroxykynurenine	0.86	+	225.1>208.0	22	10	225.1>110.0	22	16	2	5-5000	1	95	Kynurenine -D_4
p-Tyrosine	0.89	+	182.1>91.0	20	20	-	-	-	2	312-5000	5	65	Phenylalanine-D_5
m-Tyrosine	1.16	+	182.1>91.0	20	20	-	-	-	2	5-5000	3	107	Phenylalanine -D_5
Serotonin	1.45	+	177.0>115.0	20	25	177.0>160.0	20	25	10	20-5000	9	110	Serotonin-D_4
5-hydroxytryptophan	1.49	+	221.1>204.1	10	10	221.1>162.2	10	16	2	5-5000	3	97	5OHTrp-D_4
o-Tyrosine	1.51	+	182.1>91.0	20	20	-	-	-	5	10-5000	0.5	109	Phenylalanine -D_5
Kynurenine	1.62	+	209.0>94.0	20	10	209.0>146.1	10	10	2	5-5000	24	90	Kynurenine -D_4
Phenylalanine	1.68	+	166.1>91.0	20	20	-	-	-	80	80-5000	5	90	Phenylalanine -D_5
N-Formylkynurenine	1.73	+	237.1>146.1	34	22	237.1>136.0	34	16	2	5-5000	0.5	71	Kynurenine-D_4
Hydroxyanthranillic acid	2.02	+	153.9>135.9	10	15	153.9>80.0	10	25	40	80-5000	4	109	IndoleAcet-D_5
Tryptophan	2.17	+	205.0>188.0	25	10	205.0>146.0	30	20	40	80-5000	5	-	Tryptophan-D_5
Xanthurenic Acid	2.21	+	206.1>160.0	25	15	206.1>132.0	25	25	2	5-5000	5	108	Xanthurenic-D_4
Tryptamine	2.38	+	161.0>144.1	22	10	161.0>122.0	15	10	2.5	50-5000	2	108	Tryptamine-D_4
Kynurenic acid	2.39	+	190.0>88.9	10	35	190.0>144.1	10	20	2	2-5000	3	95	Kynurenic-D_5
5-Methoxytryptamine	2.52	+	191.0>159.0	20	21	191.0>143.0	20	21	2	5-5000	5	107	Tryptam-D_4
4-Chloro-kynurenine	2.64	+	243.1>226.0	10	10	243.1>128.0	10	16	5	10-5000	5	95	ClKyn-$^{13}C_2$-^{15}N
Acetylserotonin	2.68	+	219.2>160.0	10	16	219.2>115.0	10	34	2	5-5000	3	96	Tryptamine-D_4
Phenylacetylglutamine	2.75	-	265.0>145.0	20	15	265.0>127.0	20	25	2	5-5000	1	99	PAGN-D_5
6-Hydroxymelatonin	3.05	+	249.0>190.0	30	20	249.0>158.0	10	25	2	5-5000	0.5	106	OHMelatonin-D_4
Indole-3-acetamide	3.16	+	175.1>130.0	25	15	175.1>103.4	25	30	2.5	50-5000	3	106	IndoleAcet-D_5
Anthranilic acid	3.17	+	138.0>120.0	10	15	-	-	-	10	20-5000	1	111	IndoleAcet-D_5
Formyl-N-acetyl-methoxykynurenine	3.46	+	265.2>136.1	10	22	265.2>114.1	10	16	2	5-5000	1	113	ClKyn-$^{13}C_2$-^{15}N
Indolelactic acid	3.59	-	203.9>128.0	25	15	203.9>128.0	25	20	2	2-5000	9	86	Melatonin-D_4
Melatonin	3.99	+	233.2>174.1	22	16	233.3>159.1	22	28	5	10-2500	4	102	Melatonin-D_4
3-Indoleacetic acid	4.04	-	174.0>130.0	20	15	-	-	-	39	80-5000	11	78	IndoleAcet-D_5
Tryptophol	4.12	+	162.1>144.1	30	10	162.1>103.0	30	20	5	10-5000	4	98	IndoleAcet-D_5

Internal standards (ISs): Serotonin-D4 (RT: 1.44 min, MRM: 268>145, ESI+); 5-hydroxytryptophan-D_4 (5OHTrp-D_4) (RT: 1.47 min, MRM: 225>208, ESI+); Kynurenine-D_4 (RT: 1.60 min, MRM: 213>98, ESI+); Phenylalanine-D_5 (Phe-D_5)(RT: 1.67 min, MRM: 171.5>125.0, ESI+); Tryptophan-D_5 (RT: 2.15 min, MRM: 210>193, ESI+); Xanthurenic acid-D_4 (RT: 2.20 min, MRM: 210>164, ESI+); Kynurenic acid-D_5 (RT: 2.38 min, MRM: 195.0>149.0); Tryptamine-D_4 (RT: 2.38 in, MRM: 165>148, ESI+); 4-Chloro-kynurenine-$^{13}C_2$,^{15}N (ClKyn-$^{13}C_2$-^{15}N)(RT: 2.63 min, MRM: 246>128, ESI+); Phenylacetylglutamine-D_5 (RT: 2.74, MRM: 268>145, ESI-);6-hydroxymelatonin-D_4 (RT: 3.04, MRM: 253>194, ESI+); Indole-D_5-3-acetamide (IndoleAcet-D_5)(RT: 3.13 min, MRM: 180.0>134.0, ESI+); Melatonin-D_4 (RT: 3.98 min, MRM: 237>178, ESI+); LOD: Limit of detection; LR: Linear range; RSD: relative standard deviation in 3 spiked samples; Recovery: Mean recovery (n=3) in spiked samples at 500 nM.

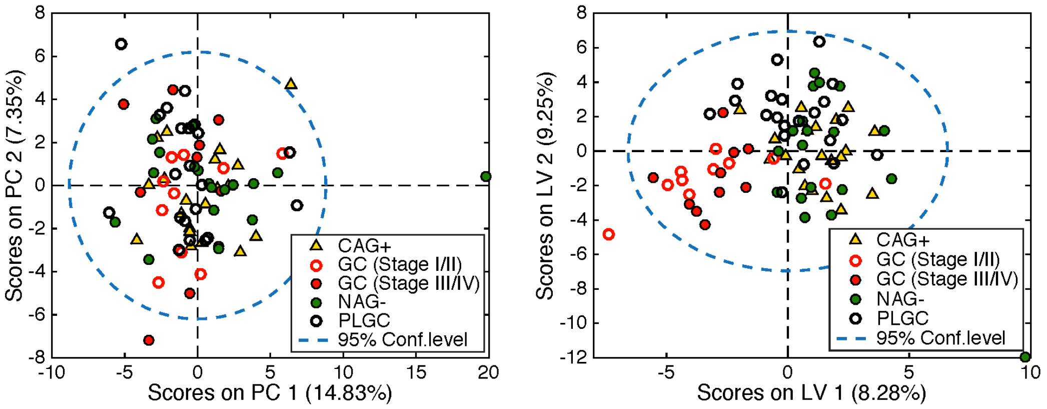

Figure S1. Multivariate analysis of metabolomic data. Scores plots from unsupervised PCA (left) and supervised PLS-DA (right) models.
